# The royal food of termites shows king and queen specificity

**DOI:** 10.1093/pnasnexus/pgad222

**Published:** 2023-07-04

**Authors:** Eisuke Tasaki, Yuki Mitaka, Yutaka Takahashi, A S M Waliullah, Zinat Tamannaa, Takumi Sakamoto, Ariful Islam, Masaki Kamiya, Tomohito Sato, Shuhei Aramaki, Kenji Kikushima, Makoto Horikawa, Katsumasa Nakamura, Tomoaki Kahyo, Mamoru Takata, Mitsutoshi Setou, Kenji Matsuura

**Affiliations:** Laboratory of Insect Ecology, Graduate School of Agriculture, Kyoto University, Kitashirakawa-Oiwakecho, Sakyo-ku, Kyoto 606-8502, Japan; Department of Biology, Faculty of Science, Niigata University, 8050 Ikarashi 2-no-cho, Nishi-ku, Niigata 950-2181, Japan; Laboratory of Insect Ecology, Graduate School of Agriculture, Kyoto University, Kitashirakawa-Oiwakecho, Sakyo-ku, Kyoto 606-8502, Japan; Department of Entomology, 2143 TAMU, Texas A&M University, 2556 F&B Rd., Building 1804, College Station, TX 77843-2143, USA; Department of Cellular and Molecular Anatomy, Hamamatsu University School of Medicine, 1-20-1 Handayama, Higashi-ku, Hamamatsu, Shizuoka 431-3192, Japan; Department of Cellular and Molecular Anatomy, Hamamatsu University School of Medicine, 1-20-1 Handayama, Higashi-ku, Hamamatsu, Shizuoka 431-3192, Japan; Department of Cellular and Molecular Anatomy, Hamamatsu University School of Medicine, 1-20-1 Handayama, Higashi-ku, Hamamatsu, Shizuoka 431-3192, Japan; Department of Cellular and Molecular Anatomy, Hamamatsu University School of Medicine, 1-20-1 Handayama, Higashi-ku, Hamamatsu, Shizuoka 431-3192, Japan; Department of Cellular and Molecular Anatomy, Hamamatsu University School of Medicine, 1-20-1 Handayama, Higashi-ku, Hamamatsu, Shizuoka 431-3192, Japan; Department of Cellular and Molecular Anatomy, Hamamatsu University School of Medicine, 1-20-1 Handayama, Higashi-ku, Hamamatsu, Shizuoka 431-3192, Japan; Department of Cellular and Molecular Anatomy, Hamamatsu University School of Medicine, 1-20-1 Handayama, Higashi-ku, Hamamatsu, Shizuoka 431-3192, Japan; Department of Cellular and Molecular Anatomy, Hamamatsu University School of Medicine, 1-20-1 Handayama, Higashi-ku, Hamamatsu, Shizuoka 431-3192, Japan; Department of Radiology, Hamamatsu University Hospital, 1-20-1 Handayama, Higashi-ku, Hamamatsu, Shizuoka 431-3192, Japan; International Mass Imaging Center, Hamamatsu University School of Medicine, 1-20-1 Handayama, Higashi-ku, Hamamatsu, Shizuoka 431-3192, Japan; Department of Cellular and Molecular Anatomy, Hamamatsu University School of Medicine, 1-20-1 Handayama, Higashi-ku, Hamamatsu, Shizuoka 431-3192, Japan; International Mass Imaging Center, Hamamatsu University School of Medicine, 1-20-1 Handayama, Higashi-ku, Hamamatsu, Shizuoka 431-3192, Japan; Department of Cellular and Molecular Anatomy, Hamamatsu University School of Medicine, 1-20-1 Handayama, Higashi-ku, Hamamatsu, Shizuoka 431-3192, Japan; International Mass Imaging Center, Hamamatsu University School of Medicine, 1-20-1 Handayama, Higashi-ku, Hamamatsu, Shizuoka 431-3192, Japan; Department of Radiology, Hamamatsu University Hospital, 1-20-1 Handayama, Higashi-ku, Hamamatsu, Shizuoka 431-3192, Japan; Department of Cellular and Molecular Anatomy, Hamamatsu University School of Medicine, 1-20-1 Handayama, Higashi-ku, Hamamatsu, Shizuoka 431-3192, Japan; International Mass Imaging Center, Hamamatsu University School of Medicine, 1-20-1 Handayama, Higashi-ku, Hamamatsu, Shizuoka 431-3192, Japan; Laboratory of Insect Ecology, Graduate School of Agriculture, Kyoto University, Kitashirakawa-Oiwakecho, Sakyo-ku, Kyoto 606-8502, Japan; Department of Cellular and Molecular Anatomy, Hamamatsu University School of Medicine, 1-20-1 Handayama, Higashi-ku, Hamamatsu, Shizuoka 431-3192, Japan; International Mass Imaging Center, Hamamatsu University School of Medicine, 1-20-1 Handayama, Higashi-ku, Hamamatsu, Shizuoka 431-3192, Japan; Department of Systems Molecular Anatomy, Institute for Medical Photonics Research, Preeminent Medical Photonics Education and Research Center, 1-20-1 Handayama, Higashi-ku, Hamamatsu, Shizuoka 431-3192, Japan; Laboratory of Insect Ecology, Graduate School of Agriculture, Kyoto University, Kitashirakawa-Oiwakecho, Sakyo-ku, Kyoto 606-8502, Japan

**Keywords:** social insect, termite, royal food

## Abstract

Society in eusocial insects is based on the reproductive division of labor, with a small number of reproductive individuals supported by a large number of nonreproductive individuals. Because inclusive fitness of all colony members depends on the survival and fertility of reproductive members, sterile members provide royals with special treatment. Here, we show that termite kings and queens each receive special food of a different composition from workers. Sequential analysis of feeding processes demonstrated that workers exhibit discriminative trophallaxis, indicating their decision-making capacity in allocating food to the kings and queens. Liquid chromatography tandem-mass spectrometry analyses of the stomodeal food and midgut contents revealed king- and queen-specific compounds, including diacylglycerols and short-chain peptides. Desorption electrospray ionization mass spectrometry imaging analyses of ^13^C-labeled termites identified phosphatidylinositol and acetyl-l-carnitine in the royal food. Comparison of the digestive tract structure showed remarkable differences in the volume ratio of the midgut-to-hindgut among castes, indicating that digestive division of labor underlies reproductive division of labor. Our demonstration of king- and queen-specific foods in termites provides insight into the nutritional system that underpins the extraordinary reproduction and longevity of royals in eusocial insects.

**Significance Statement**

Social insect colonies are composed of individuals specialized in reproduction and individuals engaged in nonreproductive tasks, where an efficient division of labor is tightly linked to food differences between castes. Termite kings and queens do not possess the gut symbionts necessary for wood digestion, and their extraordinary reproduction and longevity is achieved by feeding from workers. We have identified the food of termite kings and queens, which has been a mystery for more than 100 years, by developing food collection techniques and advanced chemical analyses. Workers fed the king and queen different food compositions, and the common ingredients included an antiaging compound acetyl-l-carnitine. This study opens new avenues to understand the nutritional aspects of the division of labor in social insects.

## Introduction

Social insects such as ants, bees, and termites have thrived by establishing a sophisticated division-of-labor system (1). They have separated the roles of reproductive and nonreproductive laboring individuals, and have increased their fecundity by specializing in each task (1, 2). Different castes have different morphology, behavior, and physiological traits (1), as well as different gene expression patterns, metabolism, chemoreception, longevity, antioxidants, and immune systems (3). Reproductive individuals of social insects overcome the trade-off between reproduction and longevity, with the most sexually active individuals being the most long-lived (4–6). A factor that makes this possible is the special food of reproductive individuals. In honeybees, royal jelly, which is fed to larvae that will become queens, has been the subject of much research (7–12) as well as socially exchanged fluids (social fluids) in other social Hymenoptera (13–15). Research on the functionality of honey bee royal jelly has not only greatly advanced the biology of honey bees (16, 17) but has also provided insights into human medicine and health sciences (18) and has been applied to food and cosmetics. However, little is known about the diet of reproductive individuals, except for honeybee queens.

Termites evolved eusociality independently of social Hymenoptera (19). The combination of their sociality with their ability to efficiently digest lignocellulose led to tremendous evolutionary success, and they have a huge impact on terrestrial ecosystems; especially in the tropics, termites account for 95% of the insect biomass in the soil (20). Unlike eusocial Hymenoptera, termite colonies have both kings and queens (21). Kings and queens completely depend on workers for feeding (22). However, the components of termite king and queen foods (royal food) have remained unknown since initial observations were made 100 years ago (23). The main reason why the topic of royal food remains a major frontier in termite biology is that it is extremely difficult to collect many physogastric kings and queens and to obtain a sufficient amount of royal food for analysis.

In this study, we established a technique for efficiently collecting termite kings and queens, and developed a method for directly sampling the royal food in sufficient quantities to perform chemical analyses. Using advanced mass spectrometry techniques, we identified king-food- and/or queen-food-specific lipids, peptides, and proteins, some of which were related to longevity and reproduction. Furthermore, mass imaging techniques revealed the transfer of cellulose-derived royal food components from workers to queens and their localization in the queens.

## Results

### King- and queen-provisioning by workers

**Fig. 1.**
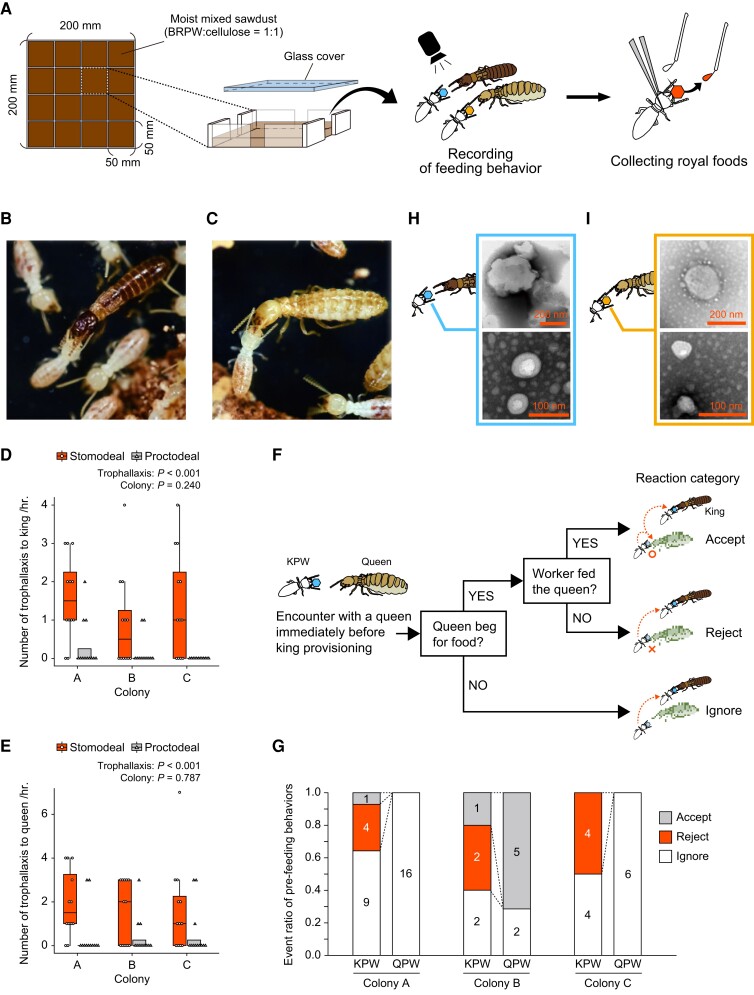
Feeding of kings and queens by workers and collection of royal food. A) Experimental setup for the observation of king and queen feeding behavior and collection of royal food. BRPW, brown rotten pine wood. B and C) Stomodeal feeding of a king B) and a queen C) by workers. D and E) Frequency of stomodeal and proctodeal trophallaxis in kings D) and queens E). *n* = 12; boxplot of the number of stomodeal and proctodeal trophallaxis per hour for each colony. Whiskers represent the lower and upper ±1.5 interquartile ranges, box edges represent the first and third quartiles, and the center line represents the second quartile (median). The data were analyzed using a GLM with a Poisson distribution. The effect of trophallaxis types (trophallaxis) and colony of origin (colony) as independent variables were examined by using the likelihood ratio test. *P*-values are shown in boxplots. F) Flow chart of reaction categories of a worker that encounters a queen immediately before king (or queen) provisioning. G) Event ratio of KPWs and QPWs in each of the three colonies. Rejection of queens was observed only by KPWs. H and I) TEM of king H) and queen food I) directly collected from provisioning workers.

What are termite kings and queens fed by workers? Are kings and queens fed different foods? To answer these questions, we analyzed the feeding behavior of the termite *Reticulitermes speratus* using a planar arena that is called a glass cell in this study. This termite has an asexual queen succession system, in which there are multiple parthenogenetically produced queens with a single king (24, 25). We conducted an experiment in which each king was placed in a glass cell lined with termite-culturing medium along with 20 queens and 250 workers. The system was video recorded for a total of 12 h. Recordings were made to determine the frequency of feeding per unit time (Fig. 1A). Trophallaxis in termites can be performed through oral and anal feeding known as stomodeal and proctodeal, respectively (26). However, both kings and queens were predominantly fed orally (Fig. 1B–E, and Movies S1 and S2). Kings and queens were fed orally by workers 1.31 ± 0.21 times per hour (mean ± SEM) and 1.72 ± 0.27 times per hour, respectively, with no significant difference between the two (likelihood ratio test, χ^2^ = 0.9426, d.f. = 1, *P* = 0.3316). However, because there was 1 king and 20 queens in the system, the total number of times queens were fed was approximately 26-fold that of each king.

To ascertain whether workers feed kings and queens differentially, we analyzed the recorded data to identify the worker's behavior immediately prior to king or queen provisioning (Fig. 1A). When a worker that eventually fed the king [king-provisioning worker (KPW)] encountered a queen, just prior to feeding the king, one of three reactions was observed: the worker also provisioned the queen (accept), the worker rejected the queen's begging (reject), or the worker passed the queen without the queen begging (ignore) (Fig. 1F). The reaction of a queen-provisioning worker (QPW) to a previous encounter with another queen was also analyzed. We observed rejection of the queen's begging by KPWs, while QPWs showed no rejection (Fig. 1G), suggesting that workers feed kings and queens differentially. To analyze the composition of royal food, we collected it directly from KPWs or QPWs (Fig. 1A; see Materials and methods, for details). Negative staining and transmission electron microscopy (TEM) of the food revealed that it consisted of ca. 50–400 nm particles (Figs. 1H and I).

### Chemical compounds of royal food

**Fig. 2.**
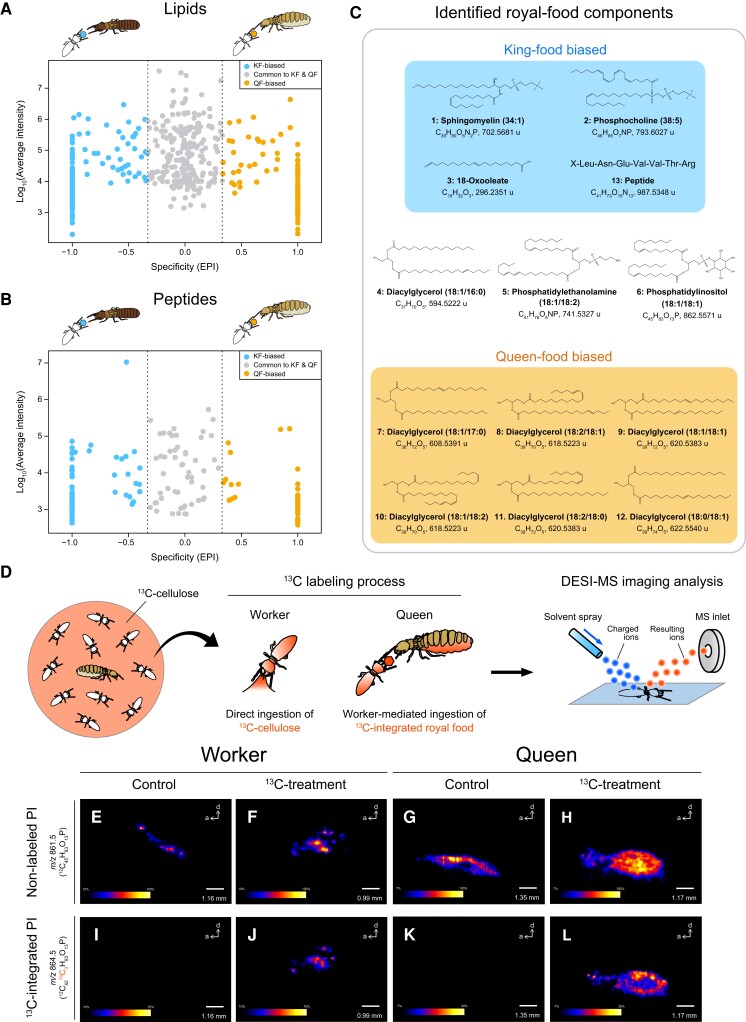
Chemical composition of king- and queen-specific foods. A and B) Lipid A) and peptide B) profiles of king- and queen-specific foods based on estimated peak intensities. Compounds that were more common in king food are indicated by plots on the left side of the figures (EPI < -1/3), and compounds that were more common in queen food are indicated by plots on the right side of the figure (EPI > 1/3). KF, king food; QF, queen food. C) Chemical components of royal food. Classification of the compounds is based on manual confirmation of peak intensities in the raw data using Xcalibur software. The positions of double bonds in each lipid are unknown. D) Tracking carbon transfer from worker to queen using ^13^C-labeled cellulose (^13^C-cellulose). E–L) Distribution of a ^13^C-integrated royal-food component, phosphatidylinositol (PI), in workers and queens by DESI-MSI. DESI-MSI of nonlabeled PI (*m*/*z* 861.5) in a control worker E), a ^13^C-cellulose-fed worker F), a control queen G), and a queen kept with ^13^C-cellulose-fed workers H). DESI-MSI of ^13^C-integrated PI (*m*/*z* 864.5) in a control worker I), a ^13^C-cellulose-fed worker J), a control queen K), and a queen kept with ^13^C-cellulose-fed workers L). a, anterior; d, dorsal.

We analyzed the lipid and peptide components of king food and queen food by liquid chromatography tandem-mass spectrometry (LC-MS/MS). We performed the same analysis on the contents of the midgut (where ingested food accumulates) in kings, queens, soldiers, and workers. We detected a total of 437 lipid species in royal food (Fig. 2A), and a total of 3,015 lipid species in the midgut contents of termites (Fig. S1A). Among the royal food components, three lipids were more abundant in king food: sphingomyelin (34:1), phosphocholine (38:5), and 18-oxooleate (Fig. 2C and Table S1). Six diacylglycerol species were more abundant in queen food (Fig. 2C and Table S1). Three lipids were almost equally abundant in both: diacylglycerol (18:1/16:0), phosphatidylethanolamine (18:1/18:2), and phosphatidylinositol (18:1/18:1) (Fig. 2C and Table S1). We detected a total of 172 peptides in royal food (Fig. 2B), and a total of 9,424 peptide species in the midgut contents of termites (Fig. S1B). We found a peptide consisting of eight amino acids (accurate mass 987.5348: X-Leu-Asn-Glu-Val-Val-Thr-Arg) mostly in king food (Fig. 2C and Table S1), although an amino acid of *N*-terminal (X) could not be determined.

In-gel proteomic analysis was performed to identify proteins specific to the midgut contents of queens (Fig. S2). Extracts of the midgut contents of queens and workers were separated by one-dimensional SDS–PAGE, revealing three queen-specific bands (α, β, and γ; Fig. S2A). These bands were excised from the gel, digested by trypsin, and analyzed for peptide fragments by LC-MS/MS, yielding 52, 55, and 11 queen-specific peptides in bands α, β, and γ, respectively (Fig. S2B–D). Next, we predicted the proteins that contain these queen-specific peptides from protein-coding sequences derived from RNA-seq data. Of the queen-specific peptides in band α, 23 were 100% identical to the amino acid sequences of 13 proteins. Multiple fragments matched arginine kinase, kynurenine–oxoglutarate transaminase 3, polyadenylate-binding protein 1, and mitogen-activated protein kinase 1 (see Fig. S2B for the other nine proteins). Of the queen-specific peptides in band β, nine were 100% identical to the amino acid sequences of eight proteins. Two fragments matched glucose-6-phosphate isomerase (see Fig. S2C for the other seven proteins). Of the queen-specific peptides in band γ, one was 100% identical to the amino acid sequence of a hypothetical protein, but no proteins with multiple fragment matches were detected (Fig. S2D).

### Tracking of carbon transfer

The royal food components would be derived from compounds ingested by workers or their metabolites because kings and queens do not forage (22). We focused on phosphatidylinositol, a royal food compound, and investigated whether the ^13^C-cellulose carbon ingested by workers is incorporated into phosphatidylinositol in queens; we used desorption electrospray ionization mass spectrometry imaging (DESI-MSI) to do this (Fig. 2D). ^13^C-integratad phosphatidylinositol was detected in the bodies of workers fed ^13^C-cellulose (Fig. 2J), and in the body of the queen provisioned by the ^13^C-cellulose-fed workers (Fig. 2L). By contrast, it was not detected in queens provisioned by workers fed nonlabeled cellulose (Fig. 2K). DESI-MSI revealed that phosphatidylinositol was particularly abundant around the fat body of the queen's abdomen (Fig. 2H and L). These results indicate that compounds produced by worker cellulose metabolism are passed on to queens as royal food.

**Fig. 3.**
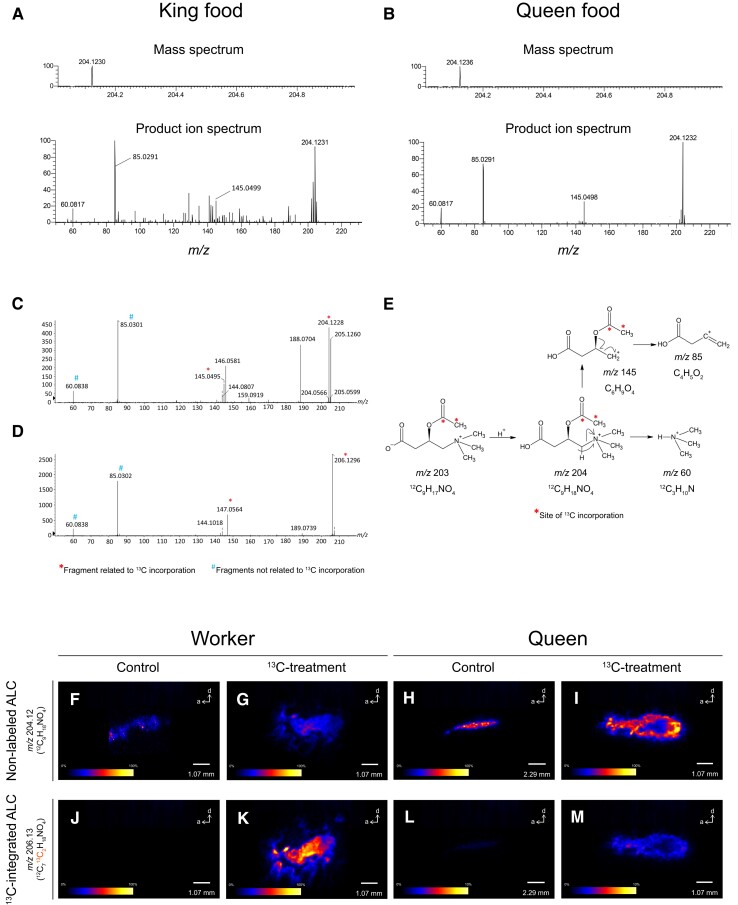
Acetyl-l-carnitine (ALC) in royal food. A and B) Mass spectra of ALC in king A) and queen food B). C and D) Product ion mass spectra of ALC in control workers C) and ^13^C-cellulose-fed workers D). E) Interpretation of fragmentation pattern and the sites of ^13^C incorporation in ALC. F–M) Distribution analysis of ALC in workers and queens by DESI-MSI. DESI-MSI of nonlabeled ALC (*m*/*z* 204.12) in a control worker F) a ^13^C-cellulose-fed worker G), a control queen H), and a queen kept with ^13^C-cellulose-fed workers I). DESI-MSI of ^13^C-integrated ALC (*m*/*z* 206.13) in a control worker J), a ^13^C-cellulose-fed worker K), a control queen L), and a queen kept with ^13^C-cellulose-fed workers M). a, anterior; d, dorsal.

Although the royal food analysis thus far has focused on lipids and peptides, important functional molecules in royal food could include low-molecular-weight compounds. One notable functional substance resulting from cellulose metabolism in termites is acetyl-l-carnitine, which has been found in the gut of workers of *Hodotermopsis sjostedti* (27). Acetyl-l-carnitine administration reportedly improves mitochondrial dysfunction associated with aging in rats (28) and prolongs the chronological lifespan of yeast by acting on its mitochondrial morphology (29). Our direct-infusion tandem-mass spectrometry analysis detected acetyl-l-carnitine in both king and queen food (Fig. 3A and B). Tracing with ^13^C-labeled cellulose confirmed that carbon derived from cellulose ingested by workers was transferred to acetyl-l-carnitine in the worker's body. The fragmentation pattern of the product ion showed that ^13^C was incorporated into the acetyl group of acetyl-l-carnitine (Fig. 3C–E). DESI-MSI was used to characterize the spatial distribution of ^13^C-integrated acetyl-l-carnitine in a queen provisioned by the ^13^C-cellulose-fed workers (Fig. 3M). Imaging revealed that acetyl-l-carnitine is particularly abundant around the fat body of the queen's abdomen (Fig. 3I and M).

### Digestive division of labor

**Fig. 4.**
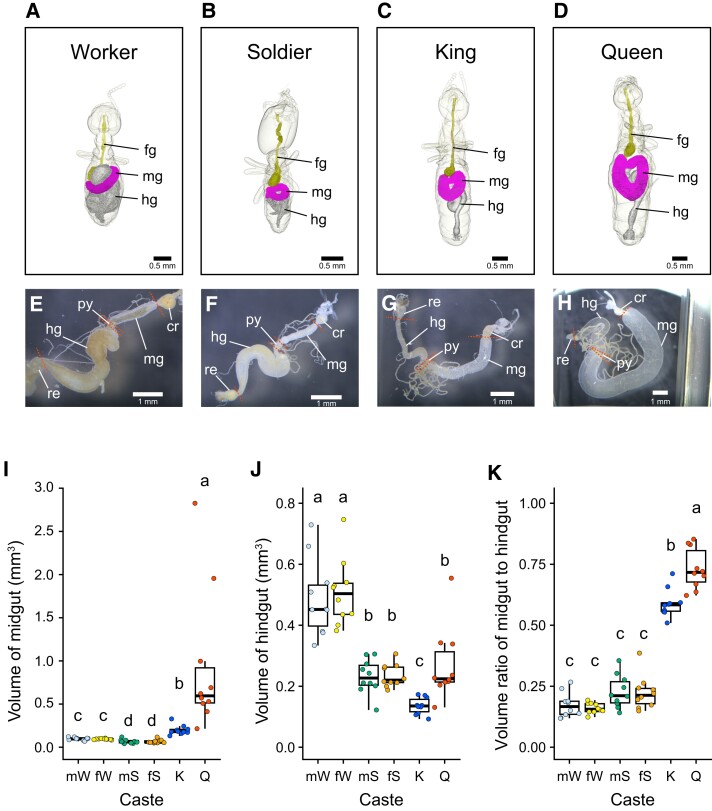
Digestive division of labor underlying reproductive division of labor. A–D) Micro-CT images of worker A), soldier B), king C), and queen D). fg, foregut; mg, midgut; hg, hindgut. E–H) Micrographs of the digestive tracts of a worker E), soldier F), king G), and queen H). cr, crop; mg, midgut; py, pylorus; hg, hindgut; re, rectum. I and J) Midgut I) and hindgut J) volumes among castes. K) Volume ratio of the midgut to hindgut. *n* = 10; boxplot of the volume of midgut and hindgut and the volume ratio for each caste. Whiskers represent the lower and upper ±1.5 interquartile range, box edges represent the first and third quartiles, and the center line represents the second quartile (median). Data were analyzed using a GLMM with gamma distributions with the gut size and the body weight as independent variables with colony of origin as a random effect using the likelihood ratio test and compared using Tukey's test for multiple comparisons. Different letters atop bars denote significant differences at *P* < 0.05. mW, male worker; fW, female worker; mS, male soldier; fS, female soldier; K, king; Q, queen.

It is conceivable that the digestive tract structure of kings and queens is specialized for the digestion and absorption of royal food, which is different from that of workers, which eat wood and digest it in the microbe-rich hindgut (30). In other words, there may be a division of digestion between reproductive and nonreproductive castes (31). Therefore, we performed between-caste comparison of the size of the midgut, the nutrient-absorbing organ, and the hindgut, the organ that holds microorganisms and performs cellulose degradation; we used micro-computed tomography (micro-CT) for this (Fig. 4). The midgut of kings and queens was larger than that of workers, whereas the hindgut of workers was larger than that of kings and queens (Fig. 4A–D). This was confirmed by morphometric measurements of gut volumes using anatomically sampled images of the digestive tract (Fig. 4E–H). The midgut volumes of the king and queen were larger than those of the worker and soldier (Fig. 4I), and the volume of the hindgut was largest in workers (Fig. 4J). Kings and queens had significantly higher volume ratios than the other castes (Fig. 4K). Intriguingly, the volume ratio of soldiers, which is dependent on caste as well as king and queen (22), was lower than that of kings and queens and comparable to that of workers, suggesting that the reproductive division of labor underlies the digestive division of labor. These results provide anatomical evidence that kings and queens are specialized for the absorption and utilization of royal food components.

## Discussion

Instead of eating wood, termite kings and queens are fed by workers and engage exclusively in reproduction. We collected royal food and analyzed its chemical composition. It would be expected that the nutritional requirements of kings and queens differ in composition and quantity because requirements for sperm production and egg production are different. We found that the king and queen foods differed in chemical composition. This is consistent with behavioral observations, in which workers feed kings and queens differentially. We found no significant difference in the frequency of stomodeal feeding between kings and queens. However, the total number of feedings to queens was higher than that to the king because a colony has many queens but a single king in asexual queen succession termites (24, 32). In higher termites, where a single physogastric queen is responsible for reproduction, the frequency of feedings to the queen would be expected to be higher than observed here.

Queen food likely contains compounds required for egg production. Vitellogenesis, the process by which vitellogenin is produced in the fat body and transported to developing oocytes, is important for reproduction in insect females (33). It is found exclusively in females among lipoproteins involved in insect lipid transport, and the major neutral lipid transported by this vitellogenin is diacylglycerol (34). We found that some diacylglycerols are more common in queen food than in king food. In insects, diacylglycerol in the diet is absorbed from the midgut lumen (35, 36), consistent with our observation that the midgut of queens is significantly larger than in other castes (Fig. 4). Lipidomic studies of the higher termite *Macrotermes natalensis* showed that the amount of diacylglycerol in queens’ fat bodies increases during reproductive development (37). Thus, it is likely that termite queens absorb diacylglycerols for egg production. These findings suggest that the termite king- and queen-specific foods (royal food) are optimized for their respective reproductive roles.

Sperm cell membranes contain sphingomyelins, which in mammals have an important role in sperm fertilization (38, 39). We found that king food contains more of this phospholipid than queen food, suggesting an important role in sperm production. In addition, dietary supplementation with sphingomyelin has been shown to benefit intestinal health and cognitive development in mammals (40–42). Reproductive termites (kings and queens) are generally extremely long-lived compared with solitary insects (4, 5). Moreover, particularly strong selection acts on the longevity of the primary kings in *R. speratus*, due to their unique reproductive system, asexual queen succession (32). Components of king food, such as sphingomyelin, might contribute to the king's extraordinary longevity. We also identified a king-food-biased peptide of unknown function (Fig. 2C). King food contains many unidentified components and substances whose functions have yet to be elucidated.

Phosphatidylinositol was detected as a lipid component in king- and queen-specific foods, and was more abundant in the midguts of kings and queens than in those of soldiers and workers (Fig. 2C). ^13^C tracking using ^13^C-cellulose and DESI-MSI (Fig. 2) demonstrated that phosphatidylinositol was biosynthesized from the cellulose ingested by workers and transferred to queens via trophallaxis. ^13^C-incorporated phosphatidylinositol was detected in the body cavity (probably in the fat body) rather than in the digestive tract. This suggests that the absorbed phosphatidylinositol is promptly transported to the fat body, where it is stored until it is used for egg production and other metabolic processes.

Acetyl-l-carnitine was identified in king and queen food (Fig. 3). Acetyl-l-carnitine is a compound associated with antiaging and longevity (28, 29). We found that ^13^C was incorporated into the acetyl group rather than carnitine in acetyl-l-carnitine (Fig. 3E), likely due to the difference in metabolic steps or metabolic rate of the acetate (as an acetyl donor) and carnitine biosynthetic pathways from cellulose. While termite workers synthesize acetate from cellulose via the metabolism of symbiotic microorganisms in the hindgut and rapidly use it for their own metabolism (30), they might use amino acids for carnitine synthesis by degrading the symbiotic microorganisms in the hindgut. Although we analyzed termite king- and queen-specific foods for the first time, the functions of many compounds are unclear. Some of the royal food components included substances used as human health foods and supplements such as sphingomyelin and acetyl-l-carnitine. The royal food database (MetaboBank accession number: MTBKS208 and MTBKS209) constructed in the present study is a gold mine of functional ingredients. There are more than 3,000 described species of termites globally, which have diverse lifestyles and reproductive patterns, and the royal food may vary among species. Using our method, it will be possible to analyze the royal foods of other termite species.

Comparison of digestive tract structure among castes revealed that the volume ratio of the midgut to the hindgut was lower in soldiers and workers compared with kings and queens (Fig. 4K). This result suggests that soldiers, to some extent, digest food that is passed to them by their nestmates through trophallaxis, although not to the same extent as workers. While soldiers are traditionally considered a dependent caste, similar to kings and queens (22), in *R. speratus*, both workers and soldiers harbor symbiotic intestinal protozoa in their hindgut, contributing to wood digestion (43). Therefore, soldiers in *R. speratus* have the potential to digest woody food and may partially contribute to food digestion, acting as part of the social stomach.

Social insects have been found to engage in the transfer of various types of materials through social interactions (13–15, 44, 45). We observed that termite kings and queens predominantly received oral feeding from workers (Fig. 1). Consequently, the royal food in termites can be classified as a form of oral trophallactic fluid, similar to the royal jelly produced by honeybee workers. Given that honeybee workers produce royal jelly from their hypopharyngeal glands (46), it is presumed that termite workers produce the royal food through secretion from their salivary glands. Based on the observed differentiation in compound composition between king food and queen food, as well as the presence of discriminative trophallaxis in workers (Fig. 1), it can be inferred that workers have the ability to make decisions regarding food allocation to kings and queens. These findings give rise to two additional hypotheses: (i) Each provisioning worker is capable of generating both types of royal food and subsequently distributing them accordingly to kings or queens. (ii) Each provisioning worker specializes in the production of either king food or queen food, contributing to the tailored provisioning of the respective royal caste. To test these hypotheses, future investigations should employ tracking analysis of provisioning workers. The investigation of social fluids in social insects has gained significant momentum, primarily focused on social Hymenoptera species. However, our study represents a notable advancement in this field by shedding light on the role and composition of social fluids in termites. By exploring the nutritional perspective, our findings contribute to a deeper understanding of the evolution of sociality in these organisms.

## Materials and methods

### Termite sampling

Twenty colonies of *R. speratus* with a primary king and secondary queens were collected in pine or Japanese cedar forests in Kyoto, Shiga, Osaka, Hyogo, and Mie, Japan, from May to September 2019–21 (Table S2).

### Feeding behavior analysis

Colony members (1 king, 20 queens, and 250 workers) were introduced into a glass cell (150 mm × 150 mm) with a 2 mm space containing termite culture medium (mixed sawdust bait (47, 48); 1:1 ratio of brown rotten pine wood [BRPW] and cellulose). After 3 days in the dark, we continuously recorded 12 filming sets (1 h filming—1 h interval) for a total of 12 h using a Raspberry Pi camera module. The king was observed in all 12 h of video, and the three randomly selected queens were observed in 4 h of video at 3 h intervals (king: 1 individual × 12 h, queen: 3 individuals × 4 h). QuickTime player (version 10.5) was used to play the video recordings to determine the frequency of stomodeal (mouth-to-mouth) and proctodeal (anus-to-mouth) feedings per unit time to the king and queen by workers. When a stomodeal event was observed, one of three reactions was observed: “Accept” means that the provisioning worker also fed another queen begging for food before feeding a king or queen. “Reject” means that the provisioning worker did not feed another queen before feeding a king or queen. “Ignore” means that the queen did not beg food from the provisioning worker. Notably, in this experiment, we did not observe any instances of QPWs rejecting the king. This lack of rejection is likely due to the higher number of queens compared with kings, resulting in a limited opportunity for the provisioning worker to encounter the king before feeding the queen. These analyses were repeated with three colonies (colonies A–C; Table S2). See Data set S1 for details.

### Royal food sampling

#### Direct sampling of king- and queen-specific foods

Two colonies were used for direct sampling of royal foods (colonies D and E; Table S2). A king and queens from the colony were introduced with workers (colony D: 1 king and 50 queens, colony E: 1 king and 46 queens) into a 2 mm thick glass cell with 16 removable glass windows (overall size: 200 × 200 mm, window size: 50 × 50 mm) containing termite culture medium. The individuals were left overnight in a condition that allowed them to freely enter and exit without drying out, so that the worker density in the glass cells was optimal. After 3 days in the dark, direct sampling was performed using stainless steel insect pin (0.3 mm diameter, Shiga Konchu Fukyusha) or filter paper (Advantec Grade No. 2, Toyo Roshi). The insect pin was bent at the tip with clean forceps; this was used for colony D. The filter paper was cut into a fan shape with clean dissecting shears; this was used for colony E. In the sampling, we opened the window of the glass cell as the worker started feeding the king or queen, pinched the feeding worker between the head and thorax with forceps, and immediately collected the food directly from between the feeding worker's mandibles, labrum, and labium. To ensure accurate sampling, if we picked a worker that had finished feeding, failed to pinch the desired spot with the forceps, or injured it with the forceps, we stopped that sampling operation and targeted another worker. In sampling using the insect pin the food sample was collected by washing the tip in analytical buffer, and in sampling with the filter paper, the food sample was collected by cutting off the tip directly into the analytical buffer. Sampling was repeated 13 times for the insect pin and 5 times for the filter paper; the samples were stored in a −80°C freezer until chemical analysis.

#### Sampling of the midgut contents of termites

Sampling of midgut contents was performed after direct feed sampling. The digestive tract of a termite individual was removed under a microscope into ice-cold phosphate-buffered saline (PBS, Thermo) and the midgut and hindgut were separated using forceps. During separation, the midgut side of the resection surface was held between the forceps to prevent the release of midgut contents into the PBS. To avoid the risk of contamination, excess PBS on the collected midgut was carefully removed with clean filter paper. Next, the resection surface of the midgut was dipped into the buffer for chemical analysis, and the contents were collected by gently pressing the midgut with forceps, which was performed with care so as not to injure the midgut tissue. These collected samples were stored in a −80°C freezer until chemical analysis.

#### Ultrastructural analysis of royal food by TEM

TEM was used to characterize the morphology of the royal food (see the section *Direct sampling of king- and queen-specific food*). Briefly, a royal food sample was smeared on an electron microscopy grid (Cu 200 mesh, Nissin EM) attached to a mesh (Excel Support Film, Nissin EM). Negative staining was performed with 1% phosphotungstic acid (PTA) for 1 min. PTA solution was passed through a 0.45 µm filter (Millex-GV, Millipore) prior to use. The grids were analyzed using a TEM (JEM-1400, JEOL).

### Chemical analysis

#### LC-MS/MS analysis

LC-MS/MS analysis of lipids was performed on an UltiMate 3000 coupled to a Q-Exactive (Quadrupole-Orbitrap Mass Spectrometer, Thermo) equipped with a heated ESI source. An Acclaim 120 C18 column (2.1 mm i.d. × 150 mm, 3 µm, Thermo) was used for chromatographic separation of termite extracts. The analysis conditions were as follows: the mobile phase flow rate was 0.3 mL/min, column temperature was 50°C, mobile phase A (water/acetonitrile/methanol = 2/1/1, 5 mM ammonium formate, 0.1% formic acid) and B (acetonitrile/2-propanol = 1/9, 5 mM ammonium formate, 0.1% formic acid), with linear gradient elution: B = 20% (0 min)−100% (50 min)–100% (60 min). The sample injection volume was 1 µL. The Q-Exactive MS was operated at a mass resolution of 70,000 (FWHM) for mass spectra and 17,500 (FWHM) for product ion spectra with a spray voltage of 3.5 kV(+)/2.5 kV(−); the S-lens RF level was set to 50. The sheath gas and auxiliary gas flow rates were set to 50 and 15 L/h, respectively. The capillary temperature and the heater temperature were set to 250 and 350°C, respectively. Mass spectra were recorded in a profile mode in a *m/z* range of 220–2,000. MS/MS was carried out in data-dependent acquisition mode with an intensity threshold of 5.0e4. The collision energy (CE) for MS/MS was set to 30 eV.

LC-MS/MS analysis of peptides were performed on an Easy-nLC 100 (Thermo) coupled with a Q-Exactive (Quadrupole-Orbitrap Mass Spectrometer, Thermo) equipped with a nano ESI source. A NTCC-360 C18 column (0.075 mm i.d. × 125 mm, 3 µm, Nikkyo Technos) was used for chromatographic separation of termite extracts. The analysis conditions were as follows: the mobile phase flow rate was 0.3 µL/min, column temperature was 50°C, mobile phase A (water, 0.1% formic acid) and B (acetonitrile, 0.1% formic acid), with linear gradient elution: B = 0% (0 min)–35% (50 min)–100% (55 min)–100% (65 min). The sample injection volume was 1 µL. The Q-Exactive MS was operated at a mass resolution of 70,000 (FWHM) for mass spectra and 17,500 (FWHM) for product ion spectra with a spray voltage of 2.0 kV(+); the S-lens RF level was set to 50. The sheath gas and auxiliary gas flow rates were set to 30 and 5 L/h, respectively. The capillary heater temperature was set to 250°C. Mass spectra were recorded in profile mode in a *m/z* range of 350–1,800 with lock-masses of *m/z* 445.1200 and 391.2842. MS/MS was carried out in data-dependent acquisition mode with an intensity threshold of 9.1e3. The CE for MS/MS was set to 27 eV.

Raw LC-MS/MS data were deposited in the DDBJ under BioProject PRJDB14286, which contains links and access to sample data through BioSample SAMD00532486–SAMD00532489 (king and queen food samples) and SAMD00532506–SAMD00532513 (Midgut content samples), and to results of analyses through MetaboBank ID MTBKS208–MTBKS209.

#### Bioinformatics analysis

To identify peptides and lipids unevenly distributed in king food or queen food, we performed a bioinformatics analysis of the LC-MS/MS results for peptides and lipids (see Supplementary Methods).

### Gel proteomics

#### Protein isolation

Protein contents from the midgut of queens and workers were collected in ice-cold buffer (20 mM Tris-HCl, 2% protease inhibitor cocktail (v/v); Nakarai tesque) using the same method as in the section, *Sampling of the midgut contents of termites*. For the three colonies (colonies F, G, and H; Table S2), protein samples were pooled for 10, 83, and 66 queens, respectively, and 50, 60, and 60 workers, respectively. These protein samples were centrifuged at 17,000 × *g*, 10 min, 4°C followed by collecting the supernatant, and the protein concentrations of each sample were determined using Bradford protein assay kit (Takara). Samples with 15 µg of protein were separated by SDS–PAGE on a 7.5% acrylamide gel followed by staining with Coomassie brilliant blue, and three gel pieces containing bands specific for the queen were cut out on a clean bench (band α, β, and γ; Fig. S2A). In addition, the gel pieces from worker sample at the same size position as the queen-specific band were also cut out and used as a control. The excised gel pieces were placed in a 1.5 mL tube, washed three times with pure water, and decolorized by adding 500 µL of 50 mM ammonium hydrogen carbonate/50% methanol followed by incubation in a 40°C heat block for 60 min. Decolorized gel pieces were washed three times again with pure water and stored in a −80°C freezer until in-gel protein digestion.

#### Protein analysis

For the in-gel protein digestion, 100 µL of reduction buffer (15 mM dithiothreitol, 25 mM ammonium hydrogen carbonate) was added to an approximately 10 × 4 × 3 mm piece of acrylamide gel, shaken at 56°C for 1 h, and the solution was discarded. The gel was washed with 100 µL of wash buffer (25 mM ammonium hydrogen carbonate) by shaking for 10 min at room temperature. Then, 100 µL of alkylation buffer (55 mM iodoacetamide, 25 mM ammonium hydrogen carbonate) was added and shaken for 45 min at room temperature in the dark. The gel was washed once and twice with 100 µL of wash buffer and 200 µL of acetonitrile: ultrapure water (1:1, v/v), respectively, and the gel piece was dried in a centrifugal concentrator. 50 µL of trypsin solution (10 µg/mL trypsin (Promega), 50 mM ammonium hydrogen carbonate) was added and left on ice for 30 min to allow the solution to penetrate the gel, and the solution remaining unabsorbed was discarded. 15 µL of 50 mM ammonium hydrogen carbonate was added, and in-gel digestion was performed at 37°C overnight (12 h or more). The next day, the trypsin solution was transferred to a tube. 50 µL of extraction buffer (acetonitrile:trifluoroacetic acid:ultrapure water, 10:1:9, v/v/v) was added to the gel and shaken at room temperature for 30 min. The extraction buffer was mixed with the transferred trypsin solution (peptide solution). The same procedure was repeated with 25 µL of extraction buffer. The peptide solution was desalted on a MonoSpin^©^ C18 column (GL Sciences): centrifugation at 2,500 × *g* for 2 min at room temperature; and 200 µL of acetonitrile, 200 µL of ultrapure water, total sample amount, 600 µL of ultrapure water, and 200 µL of acetonitrile in each step. The eluted acetonitrile solution was dried in a centrifugal concentrator, dissolved in 40 µL of 0.1% formic acid, and analyzed by LC-MS/MS in the same condition as the peptides of royal food samples.

LC-MS/MS data were analyzed using Proteome Discoverer software (version 1.4, Thermo) for peptide identification. The mass tolerance parameter was 0.01 Da for band α and β, and 0.05 Da for band γ. Mass spectra were analyzed with Thermo Xcalibur software (version 4.4, Thermo). Raw proteomic data and results of protein search (see the next section) have been deposited in the jPOST Repository (49) under the accession number JPST001871.

#### Protein prediction analysis

To determine queen-specific peptide sequences, from the data list of all identified peptide sequences (Data set S5), we picked sequences that had an observed intensity of zero in worker, an observed intensity greater than zero in queen, and were common to all three colonies (52, 55, and 11 queen-specific peptides were identified in bands α, β, and γ, respectively; See the Venn diagrams of Fig. S2B–D). To estimate the proteins with these queen-specific peptides, local BLASTP searches (50, 51) were performed against the all coding-regions in the termite RNA-seq data assembled using Trinity_v2.5.0 (52, 53) (RNA-seq data were deposited in the DNA Data Bank of Japan [DDBJ] under the BioProject PRJDB14333, which contains links and access to sample data through the BioSample SAMD00026264–SAMD00026323 and the sequence read archive DRR030795–DRR030854). Here, only estimated protein sequence with 100% identity and the same query length and alignment length were selected. These proteins containing queen-specific peptides were then annotated by performing the BLASTP searches against NCBI nonredundant protein sequences database (https://blast.ncbi.nlm.nih.gov/Blast.cgi). Moreover, we confirmed whether trypsin digestion predictions using PeptideMass (54, 55) for these protein sequences detected the same fragments as our peptide data. In those with BLASTP identity greater than 80% and the same fragments were detected in the trypsin digestion prediction (Data set S6 and Table S3), the number of peptides constituting the predicted protein was counted and visualized using a word cloud generator (https://www.wordclouds.com; Fig. S2B–D). In addition, MS/MS fragmentation annotation was performed using the two peptides constituting glucose 6-phosphate isomerase present in band β as representative sequences, and the peptide sequences obtained by LC-MS/MS analysis were confirmed to be precise (Fig. S3).

### Tracking carbon transfer using ^13^C-labeled cellulose

#### Feeding of ^13^C-labeled cellulose

A queen and 10 randomly selected workers from a colony (colony A; Table S2) were introduced into each well of a 48-well plate, in which ^13^C-labeled cellulose (^13^C-cellulose; U- ^13^C cellulose from potato [*Solanum tuberosum*] 97 atom% ^13^C, IsoLife BV) was pressed together with an appropriate amount of water. They were kept in the dark at room temperature for 1 week and then collected as ^13^C-labeled queens and workers. In addition, as the control for chemical analysis, we collected a queen and workers from two other colonies (colonies I and J; Table S2), which had been reared with termite culture medium without ^13^C-cellulose. The samples were stored in a −80°C freezer until MS analysis.

#### DESI-MSI

Ammonium formate, LC-MS grade methanol, 2-propanol, acetonitrile, and ultrapure water were purchased from Fujifilm Wako Pure Chemical Corporation. Sodium formate, leucine enkephalin, Super Cryo-Embedding Medium (SCEM), and Optimal Cutting Temperature (OCT) compound were purchased from Sigma-Aldrich, Waters, SECTION-LAB, and Sakura Finetek Japan, respectively.

The whole body of termites was embedded by SCEM in a cryo mold (Tissue-Tek Cryomold, Sakura Finetek Japan) and frozen at −20°C. The frozen termite sample was mounted on a sample holder using OCT and sectioned sagittally at 10 µm thickness at −20°C using a cryostat (CM1950, Leica Biosystems). The sections were mounted on glass slides (Matsunami) for DESI-MSI analysis.

A quadrupole time-of-flight (Q-TOF) mass spectrometer (Xevo G2-XS Q-TOF, Waters) linked to a DESI source was used to conduct the DESI-MSI study in positive and negative ion modes. Sodium formate solution (500 µM) was used at a ratio of 2-propanol:water (90:10, v/v) to calibrate the DESI mass spectra, and leucine enkephalin solution (500 µM) was used for detector set up. The solvent (98:2 methanol/water, v/v) was delivered at a flow rate of 2 µL/min using a solvent pump (Acquity UPLC Binary Solvent Manager, Waters). To achieve the best signal intensity in the tissue before acquiring data, the DESI source conditions were optimized to a capillary voltage of 4.0 kV, a nitrogen gas pressure of 0.4 MPa, an inlet temperature of 120°C, a spray impact angle of 70°, and a sampling cone of 50 V. The emitter was about 0.5 mm from the sprayer tip. The distances between the emitter tip-to-sample surface, emitter tip-to-inlet, and inlet-to-sample surface were about 2, 6, and 0.5 mm, respectively. The identified areas on the glass slide were scanned at a rate of 200 µm/s and pixel size of 100 × 100 µm. The analyzer mode was set to “sensitivity,” and the CE was set at 4.00 eV, respectively. The mass spectra from termite tissues were obtained in a *m*/*z* range of 100–1,000. To enhance mass accuracy, lock mass correction was performed using an *m/z* 281.2486 (exact *m/z* of oleic acid, deprotonated molecule) in negative ion mode and an *m/z* 309.2036 in positive ion mode.

The DESI-MSI data were acquired and processed using MassLynx software (version 4.1, Waters). HD Imaging software (version 1.4, Waters) was used to analyze ion images. The mass resolution was set to 20,000, and the mass window was 0.02 Da for peak detection in this software. The total ion current normalization function was used to normalize the DESI spectra and to produce DESI-ion images of target molecules. Raw DESI-MSI data were deposited in the DDBJ BioProject PRJDB14286, which contains links and access to sample data through the BioSample SAMD00532494–SAMD00532497 (phosphatidylinositol) and SAMD00532502–SAMD00532505 (acetyl-l-carnitine), and to results of analyses through MetaboBank ID MTBKS0212.

#### Direct infusion MS/MS analysis

Worker midgut contents were extracted in 500 µL methanol, sonicated, and centrifuged at 10,000 × *g* for 5 min at room temperature. The supernatant was collected, adjusted with 50% methanol, and 0.1% formic acid was added. Samples were analyzed by direct infusion (DI) in a Q-TOF mass spectrometer coupled to an electrospray ionization (ESI) source (TripleTOF 5600+, AB Sciex). Sample was injected at a flow rate of 1 µL/min for a total run time of 10 min. MS/MS spectra in the mass range of *m/z* 50–1,250 were obtained in positive ion mode. CE: 20.0 V was used in positive ion mode for MS/MS fragmentation of acetyl-l-carnitine. For DI-MS/MS data acquisition and analysis, Analyst TF software Build 1163 (version 1.7.1, AB Sciex) and Peakview software (version 2.1, AB Sciex) were used. Raw infusion MS/MS data were deposited in the DDBJ under BioProject PRJDB14286, which contains links and access to sample data via BioSample SAMD00532500–SAMD00532501, and to results of analyses via MetaboBank ID MTBKS211.

#### Flow injection/MS/MS analysis

King- and queen-specific foods were collected from colonies D and E (Table S2). Royal food was reconstituted in 80 µL isopropanol: acetonitrile: ultrapure water (1:1:1, v/v/v) and sonicated at 4°C for 20 min on ice. The sample was centrifuged at 94 × *g* for 5 min at 4°C. The supernatant was passed through a 0.2 μm Millex syringe-driven filter unit (Millipore).

Filtered sample was directly analyzed using flow injection (FI) in a quadrupole mass spectrometer coupled to an ESI source (Q Exactive Hybrid Quadrupole-Orbitrap Mass Spectrometer, Thermo). The instrument was connected to the Ultimate 3000 HPLC system (Thermo). Sample (5 µL) was injected at a flow rate of 200 µL/min for a total run time of 1 min. Mass spectra in a *m/z* range of 100–1,000 were obtained in positive ion mode. A higher energy collisional dissociation of 15.0 eV was used in positive ion mode for MS/MS fragmentation of acetyl-l-carnitine.

For FI-MS and FI-MS/MS data acquisition and analysis, Thermo Xcalibur software (version 4.4, Thermo) was used. The acetyl-l-carnitine's molecular assignment was implemented by considering the observed *m/z*, theoretical *m/z*, database entries (human metabolome database, LIPID MAPS) and previous studies (56, 57). Raw data were deposited in the DDBJ under BioProject PRJDB14286, which contains links and access to sample data via BioSample SAMD00532486–SAMD00532489, and to results of analyses via MetaboBank ID MTBKS210.

### Dissection and morphometric analysis

The morphologies of the digestive tracts of male and female workers, soldiers, kings, and queens were compared. Worker and soldier sexes were distinguished by the morphology of the seventh and eighth sternites following previous studies (58, 59). The head of each caste was cut off, the digestive tract was removed under a microscope into PBS, and the fat body and trachea attached to the digestive tract were carefully removed using forceps. The trimmed digestive tracts were transferred with a small amount of PBS into a Fuchs-Rosenthal chamber with a depth of 0.2 mm (Erma), and still images were taken under a microscope. If the digestive tract was damaged, measurement was stopped and redone with a new sample. The crop, midgut, pylorus (a small region at the junction of the midgut, hindgut, and Malpighian tubules), hindgut, and rectum were distinguished, and the areas of the midgut and hindgut were measured using the image-analysis software ImageJ (v2.0.0-rc-69/1.53j). The volume of each tissue was obtained by multiplying by the depth. These measurements were repeated for 10 colonies (colonies K–T; Data set S2 and Table S2). We performed micro-computed tomography (micro-CT) analysis of the morphology of the digestive tracts of the various castes (see Supplementary Methods).

### Statistics

Graphs and statistical analyses for figures were created using R software (version 4.0) and RStudio software (version 4.1). See the section *Data availability* for detailed R code information. Generalized linear model (GLM), generalized linear mixed model (GLMM), likelihood ratio test, and Tukey's test for multiple comparisons were used as indicated in the figure legends and main text. In addition, data on the number of stomodeal trophallaxes to kings and queens were analyzed by GLMM with Poisson distributions with the effects of castes (king or queen) and colony of origin as independent variables with individual ID of the king and queen as a random effect using the likelihood ratio test. *P*-values are given in the figures or in the main text. The number of biological replicates is listed in the figure legends.

## Supplementary Material

pgad222_Supplementary_Data

## Data Availability

All data are available in the main text or the supplementary materials. Raw mass spectrometry/imaging data and RNA-seq data have been deposited in the DDBJ (https://www.ddbj.nig.ac.jp/index.html). Raw proteomic data have been deposited in the jPOST repository (https://repository.jpostdb.org/). The original movie data of the workers’ feeding behaviors and raw micro-CT data may be requested from the corresponding authors (movies of feeding behaviors: Kenji Matsuura, micro-CT data: Mitsutoshi Setou). The codes for workers’ feeding behavior analysis, comparison of digestive tract structure among castes, and generation of mean-EPI plots of royal foods and heatmaps of midgut contents are all available at Github (https://github.com/eisuke-tasaki/RF_trophallaxis, https://github.com/eisuke-tasaki/gut_volume_ratio, and https://github.com/ymitaka/Analyze-termite-RF-chemical-profiles, respectively).
