# Supplementary material for: The royal food of termites shows king and queen specificity
Source: PNAS Nexus. 2023 Jul 4;2(7):pgad222. doi: 10.1093/pnasnexus/pgad222 (PMC10338896; doi:10.1093/pnasnexus/pgad222)
Supplement: pgad222_Supplementary_Data [file pgad222_supplementary_data.zip › PNASNEXUS-PNASNEXUS-2023-00231R-s01.pdf]

Supporting Information for
The royal food of termites shows king and queen specificity

Eisuke Tasaki, Yuki Mitaka, Yutaka Takahashi, A.S.M. Waliullah, Zinat Tamannaa, Takumi Sakamoto, Ariful Islam, Masaki Kamiya, Tomohito Sato, Shuhei Aramaki, Kenji Kikushima, Makoto Horikawa, Katsumasa Nakamura, Tomoaki Kahyo, Mamoru Takata, Mitsutoshi Setou, Kenji Matsuura

Corresponding author: Mitsutoshi Setou, Kenji Matsuura
Email: setou@hama-med.ac.jp (M.S.), matsuura.kenji.6s@kyoto-u.ac.jp (K.M.)

This PDF file includes:

Supporting text (Supplementary Methods)
Figures S1 to S3
Tables S1 to S3
Legends for Movies S1 to S2
Legends for Datasets S1 to S6
SI References

Other supporting materials for this manuscript include the following:

Movies S1 to S2
Datasets S1 to S6

Supporting Information Text

Supplementary Methods

Bioinformatics analysis. To discover peptides and lipids unevenly distributed in king food or queen food, we performed a bioinformatics analysis of the LC-MS/MS results for peptides and lipids.

Prior to the analysis, for the LC-MS/MS results of each sample, we exported the text file containing the list of the precursor ions that were detected in the MS2 by using QualBrowser software contained in Xcalibur™ software (version 4.4, Thermo). For each of king food and queen food samples, only the precursor ions that were common to the two colonies were extracted by using UNIX shell commands, and the list of all of the MS2-detected precursor ions that were shared between colonies and were contained in king food and/or queen food was exported as a text file (hereafter, 'MS2-detected royal food ion list').

Subsequently, we generated the list of *m/z* values of all precursor ions and their maximum peak intensities for the LC-MS/MS results of each sample. Each Xcalibur RAW MS file was converted into mzML format by using MSconvert software (1). The converted file was imported into mzMine version 2.53 (2) to detect mass of all precursor ions (parameters: Mass detector, Centroid; MS1 noise level, 5.0e0). Then, the list of *m/z* and peak intensity values of all precursor ions detected in each scan time was exported as a text file for each sample. After each of the text file was loaded into R software (version 4.0, https://www.r-project.org/), *m/z* values and intensities are rounded off to 4 and 4 decimal places, respectively, the precursor ions with duplicated *m/z* values were removed, and then the peak intensity with the highest value during the measurement time was extracted for each precursor ion. The resulting datasets were merged for peptides and lipids, respectively. Then, the precursor ions with the same *m/z* values as those on the above 'MS2-detected royal food ion list' were searched and extracted.

Although the process narrowed down the number of peptide candidates to 172, there were still 14,331 lipid candidates. Therefore, only lipids were further narrowed down to candidates. To remove the precursor ions presumed to be contaminants such as siloxanes and hydrocarbons, the percentage of the absolute value of mass defect value to the integer part of the monoisotopic mass was calculated for each lipid precursor ion, and the ions with percentages less than 0.062% and greater than 0.1% were excluded. Furthermore, to remove duplicates of the same ion that were determined to be different compounds due to *m/z* value discrepancies caused by measurement errors, only those ions with the largest average peak intensities were selected among those with duplicate *m/z* values when the *m/z* values were rounded to the second decimal place. These processes reduced the number of lipid candidates to 437. And then the resulting database (Royal food product ion database) was output as a single CSV file for peptides and lipids, respectively (Dataset S3 and S4).

To find the MS2-detected precursor ions that are biased toward king food or queen food in peptides and lipids, we calculated the mean of peak intensities of the two colonies for each of the precursor ions contained in king food and queen food (KF_{Mean} and QF_{Mean}, respectively), and an excess proportion index (EPI) was calculated for each of the precursor ions based on the following formula: $EPI = (QF_{Mean} - KF_{Mean}) / (QF_{Mean} + KF_{Mean})$. If an ion is present in queen food ($QF_{Mean} > 0$) but absent in king food ($KF_{Mean} = 0$), EPI is equal to 1. If an ion is contained in the same amount in both queen food and king food ($QF_{Mean} = KF_{Mean}$), EPI is equal to 0. If an ion is present in king food ($KF_{Mean} > 0$) but absent in queen food ($QF_{Mean} = 0$), EPI is equal to −1. We considered an MS2-detected precursor ion to be queen-food-biased if QF_{Mean} is more than twice as large as KF_{Mean} (i.e., EPI > 1/3), and king-food-biased if KF_{Mean} is more than twice as large as QF_{Mean} (i.e., EPI < −1/3). We also calculated the logarithmically transformed average of QF_{Mean} and KF_{Mean} [$\log_{10}$(Average)] for each MS2-detected precursor ion and then generated a $\log_{10}$(Average)-EPI plot. This plot was generated by using R software (version 4.0).

In parallel, the amounts of all of the MS2-detected precursor ions detected in the midgut contents were compared among castes (king, queen, soldier, and worker) for each of peptides and lipids. As with royal food data processing, we exported the text file containing the list of the precursor ions that were detected in the MS2 by using QualBrowser software contained in Xcalibur™ software (version 4.4, Thermo). The list of MS2-detected precursor ions detected in

each sample of midgut contents was combined into one and output as a text file (hereafter, 'MS2-detected midgut content ion list') by using UNIX shell commands. Subsequently, we generated the list of *m/z* values of all precursor ions and their maximum peak intensities for the LC-MS/MS results of each sample of midgut contents. Then, the precursor ions with the same *m/z* values as those on the above MS2-detected midgut content ion list were searched and extracted, and then the resulting database (Midgut content product ion database) was output as a single CSV file for peptides and lipids, respectively (Datasets S3 and S4).

Using the Midgut content product ion database, we selected the precursor ions detected from both of two colonies in the midgut contents of either caste (kings, queens, soldiers, and workers) and then calculated the mean of peak intensities of the two colonies for each of the precursor ions. After scaling the mean of peak intensities, we generated heatmap for each of peptides and lipids, respectively. The heatmap was generated by using R software (version 4.0).

Candidate peptides and lipids were predicted by using two *in silico* fragmentation tools including MS-FINDER (3) and MetFrag (4). For peptides, all of the MS2-detected compounds (king-food-biased: 74 compounds, queen-food-biased: 45 compounds, others: 53 compounds) were manually interpreted for identification. For lipids, all of the king-food- and queen-food-biased lipids (king-food-biased: 127 compounds, queen-food-biased: 82 compounds) were manually interpreted for identification, but the number of the MS2-detected lipids that were found in equal amounts in both king-food and queen-food was too large for manual interpretation (228 compounds). Therefore, based on the heatmap clustering results of lipids in midgut contents of termites, we focused only on the lipids that were detected both in midgut contents and king food/ queen food and classified as being more than twice as abundant in the midgut contents of king or queen than in the midgut contents of soldier ants or workers. In this way, we focused on 47 king-midgut-content-biased lipids and 55 queen-midgut-content-biased lipids for interpretation. Finally, the peak intensities of the focused compounds that could be identified were manually rechecked using QualBrowser software to make a final determination of whether each compound was biased toward king food and king's midgut or toward queen food and queen's midgut.

Micro computed tomography (micro-CT) analysis. The termite worker, soldier, king, and queen were put in a microcentrifuge tube and cleansed with PBS. After that, 1 mL of Bouin's solution was added and shaken overnight. After removing Bouin's solution, 1.8 mL 70% ethanol (EtOH) was added. The termites were then rinsed five times with 70% EtOH. Following that, 70% EtOH was added to the termites and shaken for 4 hours. Following that, 90% EtOH was added and shaken for 4 hours. Following that, 100% EtOH was added and shaken overnight. Then 1 mL of 2.5% (w/v) iodine in 100% EtOH was added and left stationary overnight at 4°C. Then 1.8 mL tert-butanol was added and kept overnight at 38 °C. Afterward, 100% EtOH was added and shaken for 5 min. Then 90% EtOH was added and shaken for 5 min. Then 70% EtOH was added and shaken for 5 min. Then 50% EtOH was added and shaken for 5 min. This ethanol-based approach dries the specimen and enhances contrast during the micro-CT examination. Following that, rinsed three times with PBS containing Triton-X100 (PBT). King, queen, and worker termites were separately packed inside WJM-glass capillary (3.0 mm diameter) filled with 0.5% agarose prepared with 1% PBT.

All termite samples were then scanned using a ZEISS Xradia 520 Versa (Zeiss X-ray Microscopy). The scan parameters were as follows- X-ray tube voltage, 70 kV for all samples; voxel size was 5 μm, 4.5 μm, 6 μm, and 5.9 μm for worker, soldier, king and queen, respectively. The volume data were processed using VGSTUDIO MAX software (Volume Graphics) and exported as DICOM file format. DICOM files were then imported into SYNAPSE VINCENT (version 5.3, Fujifilm). The volume-rendered images and videos were generated by manually segmenting the termite nervous system using the mask feature of SYNAPSE VINCENT software. The colors of the segmented organ were manually set.

Fig. S1

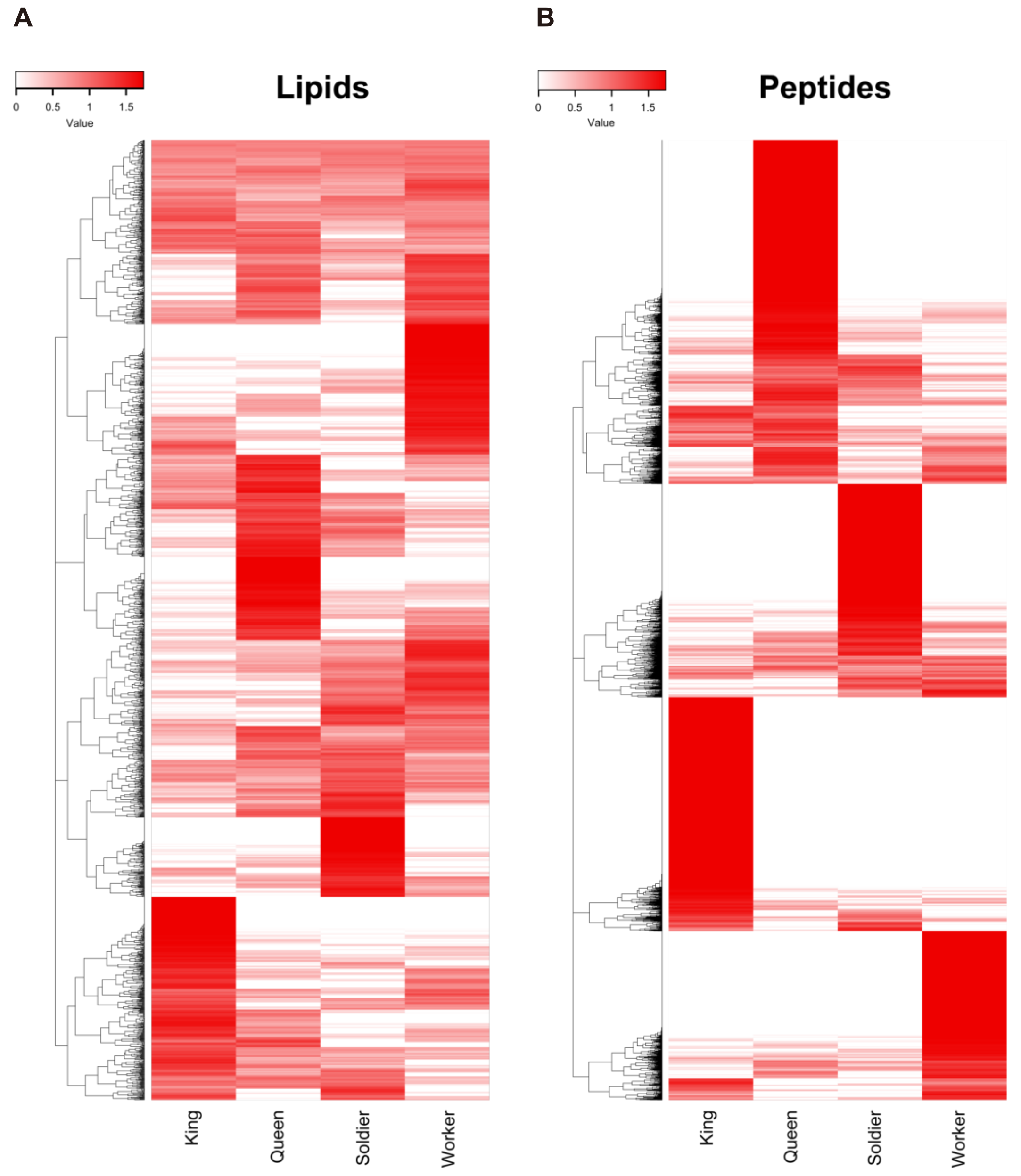

Fig. S1. Chemical compositions of midgut contents. (*A* and *B*) Comparison of lipid (A) and peptide (B) profiles among midgut contents of kings, queens, workers, and soldiers. For each component, an increase in the scaled intensity value indicates that the ion is estimated to be significantly more abundant in the midgut contents of a given caste.

Fig. S2

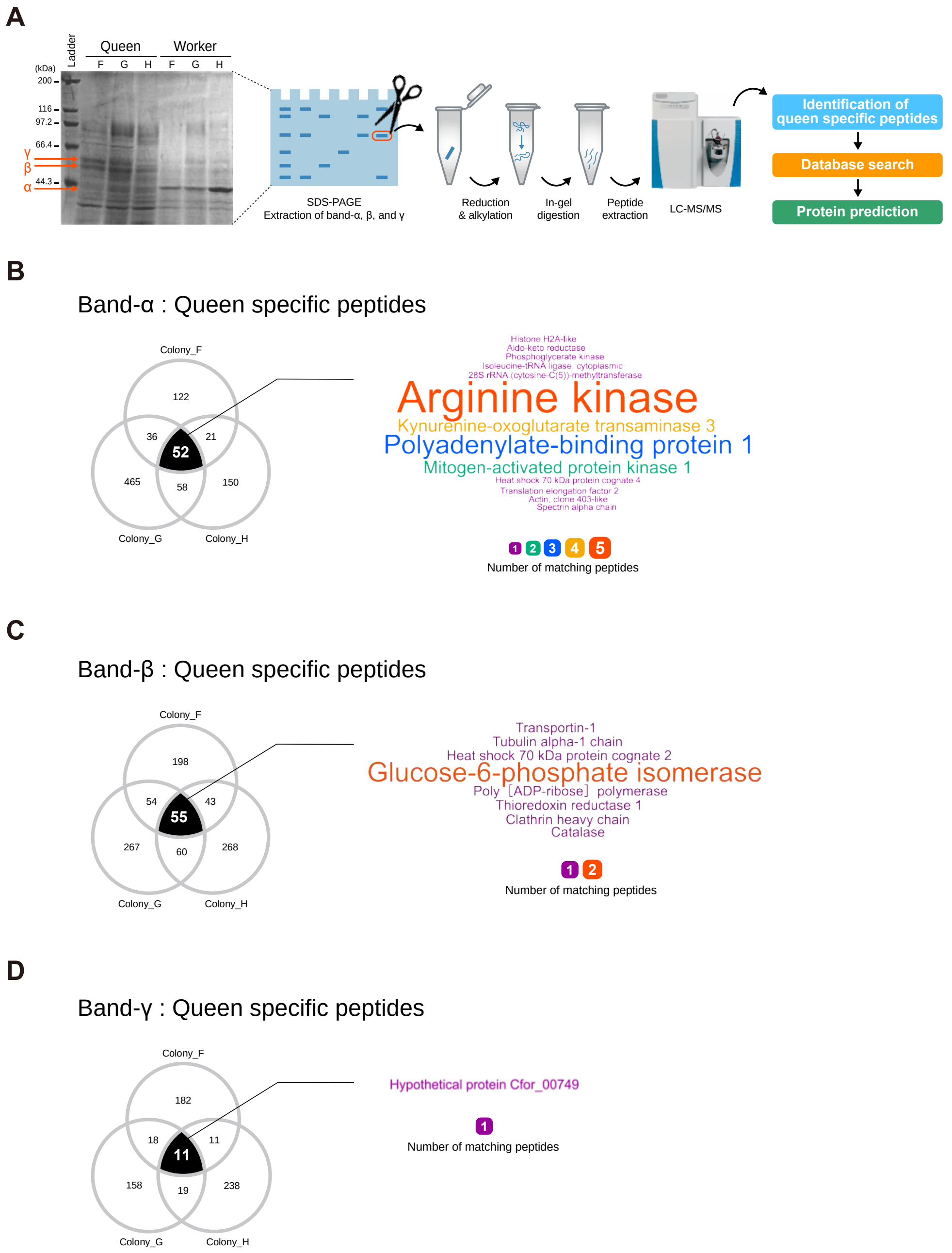

Fig. S2. In-gel proteomic analysis of queen- and worker-midgut contents. (*A*) A schematic experimental design of the search for proteins specific to the queen midgut contents. (*B* to *D*) Venn diagram analysis of queen-specific peptides in tryptic digests of the bands α (B), β (C) and γ (D). Word clouds represent the predicted proteins including the queen-specific peptides found in all three colonies; the size and colour of the word corresponds to the number of peptides mapped to the amino acid sequence of each protein.

Fig. S3

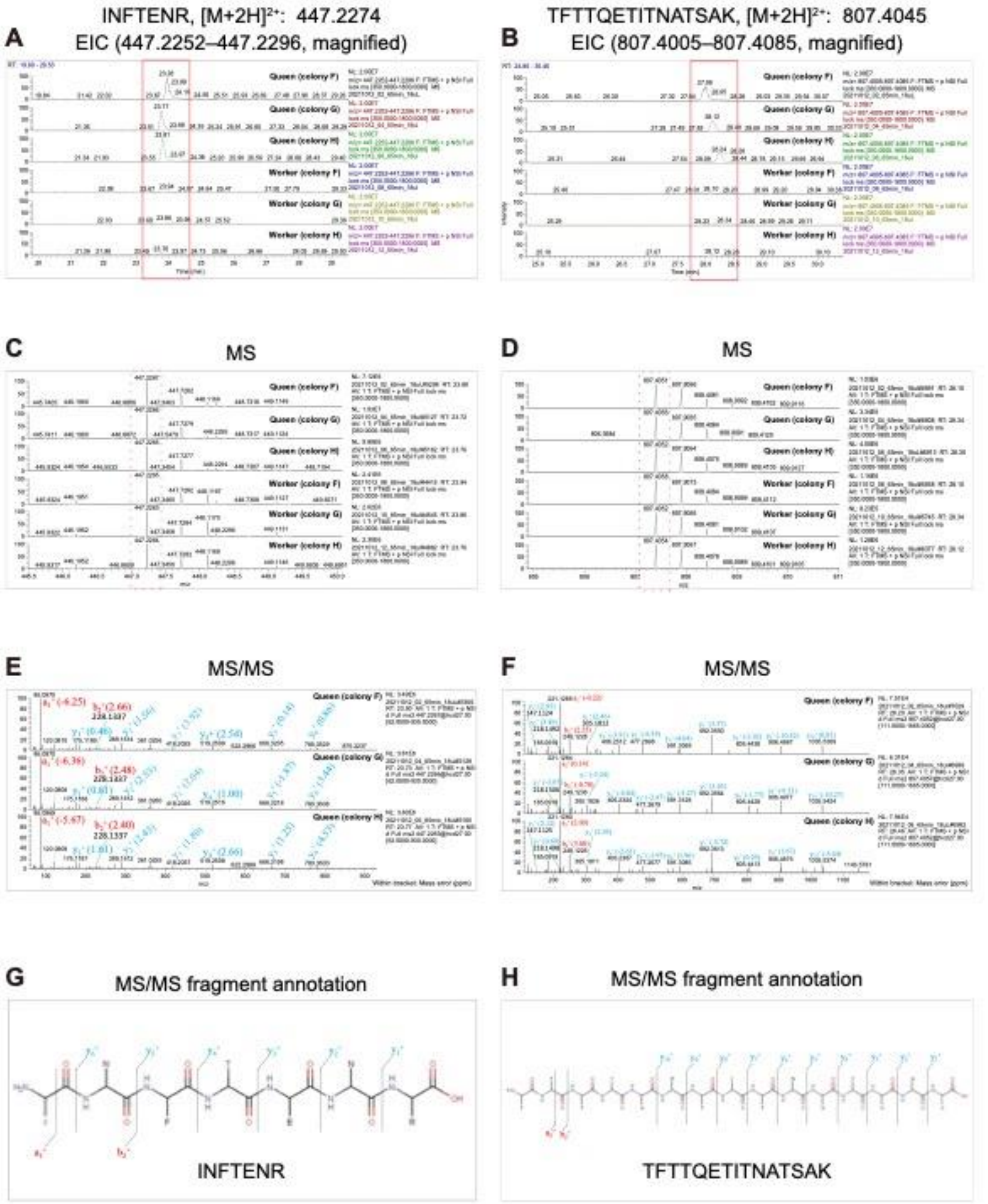

Fig. S3. LC-MS/MS analysis of queen-specific peptides constituting glucose 6-phosphate isomerase. (*A* and *B*) Extract ion chromatograms (EICs) of midgut contents from queens and workers. Two representative EICs are shown as queen-midgut-bias ions *m/z* 447.2253–447.2296 (A) and *m/z* 807.4005–807.4085 (B). Red squares show the ranges of retention time (RT) of targeted ions. (*C* and *D*) Mass spectra of target ions. Red dashed squares show monoisotopic ions. (*E* and *F*) MS/MS spectra of target monoisotopic ions. The peaks of a^+, b^+, and y^+ fragments are annotated on the MS/MS spectra of queen samples. The values in parentheses are mass errors (ppm). (*G* and *H*) “INFTENR” and “TFTTQETITNATSAK” are identified as queen-specific peptides. NL: Normalization Level.

Table S1. List of identified compounds in king- and queen-food.

Category	No.	Compound name	Molecular formula	Monoisotopic mass	*m/z* value when detected as an ion	Adduct ion	Note
Lipid	1	Sphingomyelin 34:1 (e.g., d18:0/16:1)	$C_{39}H_{80}O_6N_2P$	702.5681	705.5816	$[M + H]^+$	King-food biased compound
Lipid	2	Phosphocholine 38:5 (e.g., 18:1/20:4)	$C_{46}H_{85}O_7NP$	793.6027	794.6100	$[M + H]^+$	King-food biased compound
Lipid	3	18-Oxooleate	$C_{18}H_{32}O_3$	296.2351	297.2425	$[M + H]^+$	King-food biased compound
Lipid	4	Diacylglycerol (18:1/16:0)	$C_{37}H_{70}O_5$	594.5222	617.5120	$[M + Na]^+$	Compound in equal amount in king and queen food
Lipid	5	Phosphatidylethanolamine (18:1/18:2)	$C_{41}H_{76}O_8NP$	741.5327	764.5206	$[M + Na]^+$	Compound in equal amount in king and queen food
Lipid	6	Phosphatidylinositol (18:1/18:1)	$C_{45}H_{83}O_{13}P$	862.5571	861.5491	$[M - H]^-$	Compound in equal amount in king and queen food
Lipid	7	Diacylglycerol (18:1/17:0)	$C_{38}H_{72}O_5$	608.5391	626.5719	$[M + NH_4]^+$	Queen-food biased compound
Lipid	8	Diacylglycerol (18:2/18:1)	$C_{39}H_{70}O_5$	618.5223	641.5117	$[M + Na]^+$	Queen-food biased compound
Lipid	9	Diacylglycerol (18:1/18:1)	$C_{39}H_{72}O_5$	620.5383	643.5275	$[M + Na]^+$	Queen-food biased compound
Lipid	10	Diacylglycerol (18:1/18:2)	$C_{39}H_{70}O_5$	618.5223	636.5567	$[M + NH_4]^+$	Queen-food biased compound
Lipid	11	Diacylglycerol (18:2/18:0)	$C_{39}H_{72}O_5$	620.5383	638.5721	$[M + NH_4]^+$	Queen-food biased compound
Lipid	12	Diacylglycerol (18:0/18:1)	$C_{39}H_{74}O_5$	622.5540	640.5883	$[M + NH_4]^+$	Queen-food biased compound
Peptide	13	#X-Leu-Asn-Glu-Val-Val-Thr-Arg	$C_{41}H_{73}O_{15}N_{13}$	987.5348	494.7747 (2+)	$[M + H]^+$	King-food biased compound

#X, Undetermined amino acid.

Table S2. List of termite colonies used in this study

Colony ID	Original ID	Colony name	Royals in the colony	Collection info (y/m/d/place)	Experiments
A	TI086	190501A	PK and SQs	2019/5/1/Kyoto, Japan	Feeding behavior analysis, Tracking carbon transfer using ^{13}C-labeled cellulose
B	ET062	NA	PK and SQs	2019/7/16/Shiga, Japan	Feeding behavior analysis
C	TI129	190731D	PK and SQs	2019/7/31/Shiga, Japan	Feeding behavior analysis
D	MT552	200910A	PK and SQs	2020/9/10/Hyogo, Japan	Royal food sampling (direct feed & midgut contents)
E	MT548	200910C	PK and SQs	2020/9/10/Hyogo, Japan	Royal food sampling (direct feed & midgut contents)
F	KM079	NA	PK and SQs	2021/7/9/Mie, Japan	Gel proteomics
G	ET500	210719H	PK and SQs	2021/7/19/Kyoto, Japan	Gel proteomics
H	ET503	210719J	PK and SQs	2021/7/19/Kyoto, Japan	Gel proteomics
I	ET430	200507D	PK and SQs	2020/5/7/Kyoto, Japan	Tracking carbon transfer using ^{13}C-labeled cellulose
J	BS006	190624F	PK and SQs	2019/6/24/Osaka, Japan	Tracking carbon transfer using ^{13}C-labeled cellulose
K	MT606	210605J	PK and SQs	2021/6/18/Hyogo, Japan	Comparison of digestive tract morphology among castes
L	ET494	210610B	PK and SQs	2021/6/10/Hyogo, Japan	Comparison of digestive tract morphology among castes
M	MT609	210618A	PK and SQs	2021/6/18/Mie, Japan	Comparison of digestive tract morphology among castes
N	MT613	210618B	PK and SQs	2021/6/18/Mie, Japan	Comparison of digestive tract morphology among castes
O	MT614	210618C	PK and SQs	2021/6/18/Mie, Japan	Comparison of digestive tract morphology among castes
P	MT616	210618D	PK and SQs	2021/6/18/Mie, Japan	Comparison of digestive tract morphology among castes
Q	MT617	210618E	PK and SQs	2021/6/18/Mie, Japan	Comparison of digestive tract morphology among castes
R	MT618	210618F	PK and SQs	2021/6/18/Mie, Japan	Comparison of digestive tract morphology among castes
S	MT619	210618G	PK and SQs	2021/6/18/Mie, Japan	Comparison of digestive tract morphology among castes
T	ET497	210624A	PK and SQs	2021/6/24/Kyoto, Japan	Comparison of digestive tract morphology among castes

Table S3. Queen-specific peptide information.

Band type	Peptide ID	Query protein (Accession no.)	Blast hit protein (Accession no.)	Query organisms	E–value	Identity
Band-α	6	ICSR01000002	Kynurenine--oxoglutarate transaminase 3 (XP_023718849.1)	*Cryptotermes secundus*	0.0	91%
			Kynurenine--oxoglutarate transaminase 3 (XP_021935747.1)	*Zootermopsis nevadensis*	0.0	90%
Band-α	14	ICSR01000002	Kynurenine--oxoglutarate transaminase 3 (XP_023718849.1)	*Cryptotermes secundus*	0.0	91%
			Kynurenine--oxoglutarate transaminase 3 (XP_021935747.1)	*Zootermopsis nevadensis*	0.0	90%
Band-α	38	ICSR01000002	Kynurenine--oxoglutarate transaminase 3 (XP_023718849.1)	*Cryptotermes secundus*	0.0	91%
			Kynurenine--oxoglutarate transaminase 3 (XP_021935747.1)	*Zootermopsis nevadensis*	0.0	90%
Band-α	123	ICSR01000012	Actin, clone 403-like (XP_021922874.1)	*Zootermopsis nevadensis*	0.0	100%
Band-α	128	ICSR01000010	Heat shock 70 kDa protein cognate 4 (KDR23254.1)	*Zootermopsis nevadensis*	5E-135	94%
			Heat shock 70 kDa protein cognate 4 (XP_023722714.1)	*Cryptotermes secundus*	2E-133	95%
Band-α	154	ICSR01000002	Kynurenine--oxoglutarate transaminase 3 (XP_023718849.1)	*Cryptotermes secundus*	0.0	91%
			Kynurenine--oxoglutarate transaminase 3 (XP_021935747.1)	*Zootermopsis nevadensis*	0.0	90%
Band-α	184	ICSR01000001	Arginine kinase isoform X1 (XP_021917184.1)	*Zootermopsis nevadensis*	0.0	97%
			Arginine kinase isoform X1 (XP_023705018.1)	*Cryptotermes secundus*	0.0	96%
Band-α	186	ICSR01000011	Translation elongation factor 2 (XP_023723207.1)	*Cryptotermes secundus*	0.0	99%
			Translation elongation factor 2 (XP_021938112.1)	*Zootermopsis nevadensis*	0.0	98%
Band-α	190	ICSR01000004	Mitogen-activated protein kinase 1 (XP_021914912.1)	*Zootermopsis nevadensis*	0.0	95%
			Mitogen-activated protein kinase 1 isoform X2 (XP_023711516.1)	*Cryptotermes secundus*	0.0	95%
Band-α	274	ICSR01000013	Spectrin alpha chain isoform X4 (XP_023708714.1)	*Cryptotermes secundus*	0.0	98%
			Spectrin alpha chain (XP_021934031.1)	*Zootermopsis nevadensis*	0.0	97%
Band-α	287	ICSR01000009	probable 28S rRNA (cytosine-C(5))-methyltransferase (XP_023713329.1)	*Cryptotermes secundus*	0.0	82%
			probable 28S rRNA (cytosine-C(5))-methyltransferase isoform X1 (XP_021933348.1)	*Zootermopsis nevadensis*	0.0	81%
Band-α	331	ICSR01000001	Arginine kinase isoform X1 (XP_021917184.1)	*Zootermopsis nevadensis*	0.0	97%
			Arginine kinase isoform X1 (XP_023705018.1)	*Cryptotermes secundus*	0.0	96%

Band-α	474	ICSR01000001	Arginine kinase isoform X1 (XP_021917184.1)	*Zootermopsis nevadensis*	0.0	97%
			Arginine kinase isoform X1 (XP_023705018.1)	*Cryptotermes secundus*	0.0	96%
Band-α	564	ICSR01000001	Arginine kinase isoform X1 (XP_021917184.1)	*Zootermopsis nevadensis*	0.0	97%
			Arginine kinase isoform X1 (XP_023705018.1)	*Cryptotermes secundus*	0.0	96%
Band-α	565	ICSR01000004	Mitogen-activated protein kinase 1 (XP_021914912.1)	*Zootermopsis nevadensis*	0.0	95%
			Mitogen-activated protein kinase 1 isoform X2 (XP_023711516.1)	*Cryptotermes secundus*	0.0	95%
Band-α	627	ICSR01000003	Polyadenylate-binding protein 1 (XP_023711973.1)	*Cryptotermes secundus*	0.0	98%
			Polyadenylate-binding protein 1 (XP_021915779.1)	*Zootermopsis nevadensis*	0.0	98%
Band-α	704	ICSR01000006	Aldo-keto reductase (AFV36370.1)	*Reticulitermes flavipes*	0.0	98%
			Aldose reductase-like (XP_021940367.1)	*Zootermopsis nevadensis*	0.0	87%
			Aldo-keto reductase family 1 member B1 (XP_023711925.1)	*Cryptotermes secundus*	0.0	89%
Band-α	707	ICSR01000003	Polyadenylate-binding protein 1 (XP_023711973.1)	*Cryptotermes secundus*	0.0	98%
			Polyadenylate-binding protein 1 (XP_021915779.1)	*Zootermopsis nevadensis*	0.0	98%
Band-α	726	ICSR01000007	Phosphoglycerate kinase isoform X1 (XP_021926314.1)	*Zootermopsis nevadensis*	4E-42	94%
			Phosphoglycerate kinase isoform X1 (XP_033606549.1)	*Cryptotermes secundus*	9E-42	95%
Band-α	853	ICSR01000008	Isoleucine--tRNA ligase, cytoplasmic (XP_023710897.1)	*Cryptotermes secundus*	0.0	85%
			Isoleucine--tRNA ligase, cytoplasmic (XP_021922086.1)	*Zootermopsis nevadensis*	0.0	83%
Band-α	862	ICSR01000001	Arginine kinase isoform X1 (XP_021917184.1)	*Zootermopsis nevadensis*	0.0	97%
			Arginine kinase isoform X1 (XP_023705018.1)	*Cryptotermes secundus*	0.0	96%
Band-α	889	ICSR01000005	Histone H2A-like (XP_021917124.1)	*Zootermopsis nevadensis*	1E-90	98%
Band-α	1218	ICSR01000003	Polyadenylate-binding protein 1 (XP_023711973.1)	*Cryptotermes secundus*	0.0	98%
			Polyadenylate-binding protein 1 (XP_021915779.1)	*Zootermopsis nevadensis*	0.0	98%
Band-β	26	ICSR01000014	Glucose-6-phosphate isomerase (XP_023719146.1)	*Cryptotermes secundus*	1E-171	91%
			Glucose-6-phosphate isomerase (XP_021914062.1)	*Zootermopsis nevadensis*	2E-168	88%
Band-β	29	ICSR01000019	Thioredoxin reductase 1, mitochondrial isoform X2 (XP_023726273.1)	*Cryptotermes secundus*	0.0	92%

			Thioredoxin reductase 1, mitochondrial-like isoform X2 (XP_021918714.1)	*Zootermopsis nevadensis*	0.0	88%
Band-β	91	‡FX983163	Catalase (XP_023702049.1)	*Cryptotermes secundus*	0.0	92%
			Catalase (XP_021915774.1)	*Zootermopsis nevadensis*	0.0	91%
Band-β	463	ICSR01000020	Clathrin heavy chain isoform X1 (XP_021913695.1)	*Zootermopsis nevadensis*	0.0	99%
			Clathrin heavy chain (XP_023713342.1)	*Cryptotermes secundus*	0.0	98%
Band-β	471	ICSR01000015	Transportin-1 isoform X2 (XP_021941357.1)	*Zootermopsis nevadensis*	1E-89	98%
			Transportin-1 (XP_023724249.1)	*Cryptotermes secundus*	6E-89	97%
Band-β	522	ICSR01000014	Glucose-6-phosphate isomerase (XP_023719146.1)	*Cryptotermes secundus*	1E-171	91%
			Glucose-6-phosphate isomerase (XP_021914062.1)	*Zootermopsis nevadensis*	2E-168	88%
Band-β	543	ICSR01000016	Tubulin alpha-1 chain (PNF20591.1)	*Cryptotermes secundus*	9E-104	100%
Band-β	673	ICSR01000018	Poly [ADP-ribose] polymerase isoform X2 (XP_023719719.1)	*Cryptotermes secundus*	0.0	82%
			Poly [ADP-ribose] polymerase isoform X1 (XP_023719718.1)	*Cryptotermes secundus*	0.0	82%
Band-β	674	ICSR01000017	Heat shock 70 kDa protein cognate 2 (XP_023726137.1)	*Cryptotermes secundus*	0.0	97%
			Heat shock 70 kDa protein cognate 2-like (XP_021939718.1)	*Zootermopsis nevadensis*	0.0	94%
Band-γ	488	ICSR01000021	Hypothetical protein Cfor_00749 (GFG37055.1)	*Coptotermes formosanus*	0.0	95%

Movie S1 (separate file). Representative orally feeding behavior (stomodeal trophallaxis) in a king. See the MOV file "PNASNEXUS-PNASNEXUS-2023-00231R-s02.mov"

Movie S2 (separate file). Representative orally feeding behavior (stomodeal trophallaxis) in a queen. See the MOV file "PNASNEXUS-PNASNEXUS-2023-00231R-s03.mov"

Dataset S1 (separate file). Data set of trophallaxis behavior analysis used in this study. Colony, colony information used in this analysis. PK trophallaxis, data set of the trophallaxis behavior focused on a king. SQ trophallaxis, data set of the trophallaxis behavior focused on queens. See the EXCEL file "PNASNEXUS-PNASNEXUS-2023-00231R-s04.xlsx"

Dataset S2. (separate file). Data set of comparison of digestive tract structure among castes. See the EXCEL file "PNASNEXUS-PNASNEXUS-2023-00231R-s05.xlsx"

Dataset S3. (separate file). Data sets of the comparison of lipid profile between king food and queen food and among midgut contents of kings, queens, soldiers, and workers. See the EXCEL file "PNASNEXUS-PNASNEXUS-2023-00231R-s06.xlsx"

Dataset S4. (separate file). Data sets of the comparison of peptide profile between king food and queen food and among midgut contents of kings, queens, soldiers, and workers. See the EXCEL file "PNASNEXUS-PNASNEXUS-2023-00231R-s07.xlsx"

Dataset S5. (separate file). List of peptides predicted by in-gel proteomics: Band-α, -β, and -γ, list of peptides found from band α, β, and γ, respectively. See the EXCEL file "PNASNEXUS-PNASNEXUS-2023-00231R-s08.xlsx"

Dataset S6. (separate file). List of results of trypsin digestion predictions. See the EXCEL file "PNASNEXUS-PNASNEXUS-2023-00231R-s09.xlsx"

SI References

1. M. C. Chambers, *et al.*, A cross-platform toolkit for mass spectrometry and proteomics. *Nat. Biotechnol.* **30**, 918–920 (2012).

2. T. Pluskal, S. Castillo, A. Villar-Briones, M. Oresic, MZmine 2: modular framework for processing, visualizing, and analyzing mass spectrometry-based molecular profile data. *BMC Bioinformatics* **11**, 395 (2010).

3. H. Tsugawa, *et al.*, A cheminformatics approach to characterize metabolomes in stable-isotope-labeled organisms. *Nat. Methods* **16**, 295–298 (2019).

4. C. Ruttkies, E. L. Schymanski, S. Wolf, J. Hollender, S. Neumann, MetFrag relaunched: incorporating strategies beyond in silico fragmentation. *J. Cheminform.* **8**, 3 (2016).
